# Is there a relationship between diet quality and bone health in elderly women? A cross-sectional study

**DOI:** 10.20945/2359-3997000000394

**Published:** 2021-09-29

**Authors:** Silvia Andréa Destefani, Cilmery Suemi Kurokawa, Sérgio Augusto Rodrigues, José Eduardo Corrente, Carlos Roberto Padovani, Sérgio Alberto Rupp de Paiva, Gláucia Maria Ferreira da Silva Mazeto

**Affiliations:** 1 Universidade Paulista Departamento de Nutrição Bauru SP Brasil Departamento de Nutrição, Universidade Paulista (Unip), Bauru, SP, Brasil; 2 Universidade Estadual Paulista Faculdade de Medicina de Botucatu Departamento de Pediatria Botucatu SP Brasil Departamento de Pediatria, Faculdade de Medicina de Botucatu, Universidade Estadual Paulista (Unesp), Botucatu, SP, Brasil; 3 Universidade Estadual Paulista Faculdade de Ciências Agronômicas Botucatu SP Brasil Faculdade de Ciências Agronômicas, Universidade Estadual Paulista (Unesp), Botucatu, SP, Brasil; 4 Universidade Estadual Paulista Instituto de Biociências Departamento de Bioestatística Botucatu SP Brasil Departamento de Bioestatística, Instituto de Biociências, Universidade Estadual Paulista (Unesp), Botucatu, SP, Brasil; 5 Universidade Estadual Paulista Faculdade de Medicina de Botucatu Departamento de Clínica Médica Botucatu SP Brasil Departamento de Clínica Médica, Faculdade de Medicina de Botucatu, Universidade Estadual Paulista (Unesp), Botucatu, SP, Brasil

**Keywords:** Bone density, bone remodeling, diet, diet records

## Abstract

**Objective:**

To evaluate whether there is a relationship between diet quality and bone health in a group of elderly Brazilian women.

**Subjects and methods:**

A cross-sectional study was performed with 105 elderly women. Participants were evaluated regarding diet quality (good, needing improvement, and poor) and its relationship with bone mineral density (BMD), bone-specific alkaline phosphatase (BSAP), and C-telopeptide (CTX).

**Results:**

Fifty eight participants (55.2%) presented a poor-quality diet and 47 (44.8%) required dietary improvements, while no subjects presented a good quality diet. The group requiring dietary improvements had lower CTX [0.35 (0.05;1.09) *vs.* 0.52 (0.10;1.45); p = 0.03)] and BSAP (38.7 ± 12.9 U/L *vs.* 46.10 ± 15.2 U/L; p < 0.01) levels than the poor-quality diet group. Groups did not differ in terms of BMD.

**Conclusion:**

In this group of elderly Brazilian women, there was a relationship between diet quality and bone health, where worse diet quality was associated with higher levels of bone remodelling markers.

## INTRODUCTION

The worldwide elderly population is increasing ( 1 ). Ageing, although a natural process in life, is accompanied by chronic disabling changes which impact health and quality of life ( 2 ). These changes result from imbalances in both organofunctional and behavioural components, of which dietary intake stands out for having repercussions on the nutritional state of individuals ( 3 ).

Bone loss is a disorder related to ageing, possibly resulting in osteoporosis ( 2 ), which is a worldwide public health problem mainly affecting postmenopausal women. In fact, one in two over 50 years old women can present the disease ( 4 ), which is associated with an increased risk of fractures which considerably impact patient life quality and expectancy ( 4 , 5 ). Thus, preventing this medical condition by maintaining bone integrity is one of the main objectives when talking about health in the elderly, and, particularly, in elderly women.

The relationship between diet and bone has been studied by various authors. Mineral and other nutrient intake have been cited as potentially related to bone health ( 6 , 7 ). It was initially believed that specific nutrients, particularly calcium and phosphorous found in foods, were uniquely responsible for the diet-bone health relationship, as both are substrates for the formation of bone tissue. However, even in countries where calcium intake is within recommended levels, elevated osteoporosis rates have been observed, suggesting that other factors, which could include other nutritional aspects, may influence bone health ( 8 - 10 ). Also, a sufficient intake of proteins, vitamins D and K, among other nutrients found in dairy products, fruit and vegetables, seem to promote a more alkaline physiological environment favourable to bone metabolism ( 2 , 7 ). In fact, certain dietary standards seem to be associated with bone mineral density (BMD) ( 8 - 10 ), and some other foods, not only the dairy derivatives, have been associated with reduced bone reabsorption and risk of osteoporosis in post-menopause women ( 11 , 12 ). In this context, adequate intake, not only of isolated nutrients, but of nutrient combinations with synergistic actions, found in specific food groups, and the quality of the diet itself, emerge as influencers of bone health.

Diet quality has been evaluated using different tools depending on the focus of the study ( 13 , 14 ). Regardless of the instrument used, better quality diets are associated with higher BMD in adult women ( 15 ), and lower fracture risk in postmenopausal women ( 16 ). However, despite the relationship existing between bone health and this nutritional parameter ( 17 ), and the risk of adverse skeletal outcomes to which the elderly female population is exposed, there are few reports evaluating this association in older women, particularly employing bone remodelling markers ( 18 ), and in the Brazilian population. Thus, this study aimed to assess whether there is a relationship between bone health and diet quality, in a group of elderly Brazilian women.

## SUBJECTS AND METHODS

### Study design

This cross-sectional study evaluated a group of elderly Brazilian women for general, dietary, and densitometric characteristics, and for bone remodelling markers. Each patient was submitted to two evaluations 30 days apart: the first collecting data on general and dietary characteristics, and the second, 30 days later, repeating the first evaluation plus collecting blood samples for biochemical analysis, and evaluating bone density. Data were collected between February and November 2011.

### Participants

**Figure 1:**
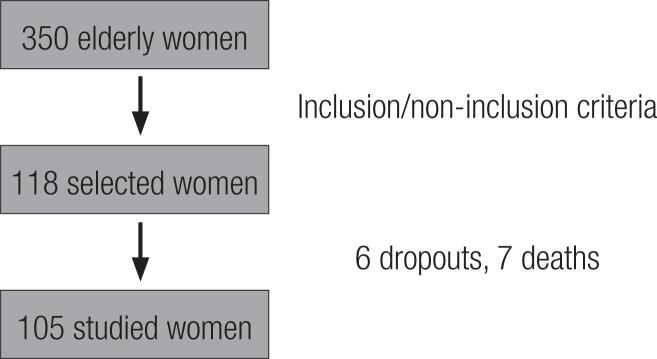
Flowchart with the study participants.

We initially evaluated 350 women aged over 60 attending an outpatient clinic specialized in elderly people located in the city of Bauru, São Paulo state, Brazil. Those with the following diagnoses were not included: decompensated diabetes, severe arterial hypertension, chronic renal insufficiency, hepatopathies, sequelae from cerebral vascular accident, protein-energy undernutrition, cancer, hyperparathyroidism, hyperthyroidism, or severe chronic obstructive pulmonary disease. Also excluded were those undergoing antiresorptive bone therapy, corticoid therapy, calcium or vitamin D supplementation, recombinant parathyroid hormone therapy, or taking any other medication known to affect bone metabolism, up to one year prior to the start of the study. In cases where obtaining data from the patient was difficult or unreliable, the caregiver’s assistance was requested. Sample size was calculated taking into account that for linear correlations of at least 0.25 to be considered significant it is necessary to evaluate a sample with 101 individuals, to attain 95% confidence and 75% test power. Considering these and the inclusion criteria, one hundred and five individuals were studied ( Figure 1 ).

### General data

The general characteristics evaluated were age (in years), race, history of smoking, alcoholism and fractures, weight (in kg), height (in m), and body mass index (BMI – in kg/m^2^). Body weight was measured using a Filizola^®^ platform scales graduated in 100 g steps and a maximum weight of 300 kg, with the patient standing upright in light clothing and no shoes. Height was measured by vertical stadiometer of the same manufacturer, attached to the scales, marked in millimetres with a 0.5 cm scale, with the patient instructed to keep her arms by her side, feet together, and take and hold a deep breath. BMI was calculated using weight and height applying the Quetelet index (weight/height^2^).

### Diet quality evaluation

Dietary intake was evaluated by calculating the average between two 24-hour recalls (R24H), always applied by the same interviewer (S.A.D.), 30 days apart, from Tuesday to Friday. This way, we attempted to verify habitual and recent nutrient intake ( 19 - 21 ). When a patient demonstrated difficulty in sizing a portion or utensil, Digital Food Photography for Dietary Surveys methodology was used ( 22 ). The use of recalls can be subject to intra-individual variations which can be minimised adjusting nutrient by total energy intake as well as applying this instrument on representative days of the different year seasons ( 19 - 21 ), as performed in this study.

The data obtained from the R24H were used to evaluate food consumption by food groups ( 23 ), including sodium, trans fats, and calories from solid fat, alcohol and added sugar intake. Quantification of these dietary components provided diet quality assessment through the Diet Quality Index-Revised (DQI-R), validated for the general population ( 24 ), and the Healthy Eating Index (HEI) ( 25 ). According to this index, diet can be classified as good quality when scoring more than 80, needing improvement between 51 and 80, and poor when less than 50.

### Dual energy X-ray absorptiometry measurements

BMD was evaluated by bone densitometry (BD) using DXA (Dual X-Ray Absorptiometry) and Lunar bone densitometry equipment (GE Healthcare, Madison, WI, USA). We measured the femoral neck (FN), whole femur (WF) and lumbar spine (LS), having calculated BMD in g/cm^2^, and standard deviation (SD) in relation to the young adult (T score), in these sites. The coefficients of variation (CV) were 1.9% for LS, 1.8% for WF, and 2.5% for FN. Osteoporosis was considered when patients presented a BMD T score ≤ -2.5, osteopenia when between -1.01 and -2.49, or normal BD when > -1.0 SD ( 26 ). Densitometric diagnosis of osteoporosis or osteopenia was based on the observation of low BMD in any of the evaluated sites, even without a history of osteoporotic fracture.

### Bone remodelling markers

The markers used to measure bone remodelling were bone specific alkaline phosphatase (BSAP), which reflects bone formation, and C-telopeptide or the carboxy-terminal portion of procollagen I (CTX), which reflects bone resorption, both analysed by serum concentration ( 27 ). Blood was collected from fasting patients and centrifuged, frozen and stored at -20 °C for later analysis. Serum BSAP measurement was performed by immunoenzyme assay using the BAP EIA kit (Metra Biosystems Inc, Mountain View, CA, USA), with reference values for over 55 years old women varying from 14.8 to 43.4 U/L, and with intra- and inter-assay CV of 8 and 7.6%, respectively. Serum CTX measurement was performed by the immunoelectrochemical method (β-CrossLaps/serum, Roche Diagnostics GmbH; using ELECSYS apparatus – ROCHE TM; Mannheim, Germany), with reference values for post-menopause women between 0.104 and 1.008 ng/mL, and inter- and intra-assay CV of 5% and 4.6%, respectively. According to these markers, serum levels of bone remodelling markers were classified as normal, increased, or reduced.

This study was approved by the Research Ethics Committee of Botucatu Medical School (Of. 471/08 and protocol no. 4326-2012). The research was executed in accordance with the Helsinki Declaration of 1975, as revised in 2008. All subjects gave their informed consent for inclusion before participating in the study.

### Statistical analysis

Collected data were entered into an Excel^®^ spreadsheet (Microsoft, USA) for later analysis. Initially, variables were described as a percentage, when categorical, and as mean, standard deviation, median, and minimum and maximum values, or first and third quartiles when numerical. When comparing two groups, numerical values were analysed using the Student’s t test in the case of normal distribution, and the Mann-Whitney’s test when there was an asymmetric distribution. Racial distribution was analysed using the Chi-square test, and comparisons between dosage category proportions for each marker were made by simultaneous 95% confidence intervals according to Goodman. Spearman correlation was used to evaluate the existence of an association between diet groups and bone markers. We used livreR-Gui software for data analysis ( 28 ). Significance level was set at 5%.

## RESULTS

The study group was characterised by presenting mean (± standard deviation) values for age of 67.5 (±7.4) years, weight of 70.8 (±12.4) kg, height of 1.57 (±0.07) m, and BMI of 28.5 (±4.8) kg/m². Seventy-nine patients (75.2%) referred to be white ( Table 1 ). There were no reports of alcoholism, smoking, or history of fractures. None of the study individuals presented a good quality diet: 58 (55.2%) presented a poor-quality diet, and 47 (44.8%) required improvements. Twenty-three (22%) individuals presented osteoporosis and 46 (44%) osteopenia, with 36 (34%) presenting normal BD. Serum BSAP levels were elevated or normal in most individuals (p < 0.05) while 94.3% presented normal CTX levels (p < 0.05; Table 2 ).

**Table 1:** Characteristics of the studied population

Bone remodeling markers	Groups		
	General n = 105	Needing improvements n = 47	Bad quality n = 58
Age (years) *	67.5 ± 7.4	68.0 ± 7.7	67.0 ± 7.2
White referred race n (%) **	79 (75.2)	37 (78.7)	42 (72.4)
Weight (kg) *	70.8 ± 12.4	70.7 ± 11.2	70.9 ± 13.3
Height (m) *	1.57 ± 0.07	1.58 ± 0.06	1.56 ± 0.08
BMI (kg/m²) *	28.5 ± 4.8	28.2 ± 4.2	28.8 ± 5.2

BMI: body mass index; kg: kilos; kg/m²: kilograms per square meter; m: meter; n: number of subjects; %: percentage of subjects.

* Mean ± standard deviation, Kruskal-Wallis non-parametric analysis of variance complemented with Dunn’s multiple comparison test.

** Chi-square test. There was no significant difference between groups (p > 0.05).

**Table 2:** Serum levels of bone remodeling markers

Bone remodeling markers	Serum levels		
	Normal	Low	High
	n (%)	n (%)	n (%)
BSAP	60 (57.1)^b^	2 (1.9)^a^	43 (41.0)^b^
CTX	99 (94.3)^b^	2 (1.9)^a^	4 (3.8)^a^

Goodman’s test. Different lowercase letters indicate significant differences between the proportions of categories within each marker (b>a; p-value < 0.05). BSAP: bone-specific alkaline phosphatase; CTX: C-telopeptide of type 1 collagen; n: number of subjects; %: percentage of subjects.

When evaluating food intake by food group, we observed that the poor-quality diet group ingested less milk/dairy products, vegetables, fruits/juices, and oils, fats and seeds than the requiring improvements group (p < 0.01; Table 3 ).

**Table 3:** Diet quality and ingestion of food groups

Food groups (number of portions)	Diet quality		p-value
	Needing improvements n = 47	Bad quality n = 58	
Cereals	2.95 (1.22; 7.75)	2.83 (0.70; 6.50)	0.34
Legumes	0.45 (0.00; 1.70)	0.25 (0.00; 3.75)	0.22
Fruits/Juices	1.55 (0.00; 5.75)	1.00 (0.00; 5.80)	<0.01
Vegetables	1.00 (0.00; 3.20)	0.50 (0.00; 2.40)	<0.01
Milk/Dairy products	1.70 (0.00; 5.05)	1.10 (0.00; 3.40)	<0.01
Meat/Eggs	1.20 (0.30; 3.35)	1.15 (0.00; 4.55)	0.88
Oils/Fats/Seeds	1.00 (0.00; 2.50)	0.50 (0.00; 2.50)	<0.01
Sweets/Added sugars	0.00 (0.00; 2.00)	0.25 (0.00; 4.25)	0.13

Results are presented as median (minimum, maximum) values, Mann-Whitney’ test (significant p-value < 0.05).

Individuals in the poor-quality diet group presented much higher serum levels of CTX and BSAP than those in the requiring improvements group (p < 0.05; Table 4 ). Milk/dairy products consumption negatively correlated with serum BSAP levels (r = -0.20, p = 0.04), while vegetables positively correlated with CTX levels (r = 0.21, p = 0.03). No other significant correlations were observed between the other food groups and remodelling markers ( Table 5 ).

**Table 4:** Diet quality, bone mineral density, and remodeling markers

Bone mineral density/Remodeling markers	Diet quality		p-value
	Needing improvements n = 47	Bad quality n = 58	
BMD-L1-L4 (g/cm²) *	1.02 (0,93-1.13)	1.04 (0.97-1.19)	0.28
BMD-TF (g/cm²) *	0.89 (0.83-0.98)	0.87 (0.80-1.07)	0.93
BMD-FN (g/cm²) *	0.82 (0.75-0.92)	0.82 (0.73-0.98)	0.91
BSAP (U/L) ‡	38.70 ± 12.90	46.10 ± 15.20	<0,01
CTX (ng/mL) *	0.35 (0.24-0.55)	0.52 (0.33-0.66)	0.03

* Median (quartile 1-quartile 3), Mann-Whitney’ test.

‡ Mean ± standard deviation, Student’s test.

Significant p-value < 0.05. BMD-FN: bone mineral density of femoral neck; BMD-L1-L4: bone mineral density of lumbar spine; BMD-TF: bone mineral density of total femur; BSAP: bone-specific alkaline phosphatase; CTX: C-telopeptide of type 1 collagen; g/cm²: grams per square centimeter; ng/mL: nanogram per milliliter; U/L: unit per liter.

**Table 5:** Correlation * between food groups and bone mineral density/remodeling markers

Food groups	Bone mineral density						Bone remodeling markers			
	BMD-L1_L4		BMD-TF		BMD-FN		BSAP		CTX	
	r	p-value	r	p-value	r	p-value	r	p-value	r	p-value
Cereals	-0.02	0.80	-0.12	0.24	-0.06	0.54	-0.07	0.46	-0.16	0.10
Legumes	-0.02	0.81	-0.09	0.34	-0.12	0.22	0.04	0.67	0.01	0.93
Fruits/Juices	0.01	0.94	-0.01	0.95	0.04	0.69	0.03	0.73	0.03	0.72
Vegetables	-0.12	0.22	-0.12	0.21	-0.13	0.20	0.13	0.18	**0.21**	**0.03**
Milk/Dairy products	-0.03	0.80	-0.03	0.75	-0.08	0.41	**-0.20**	**0.04**	-0.16	0.11
Meat/Eggs	0.16	0.11	0.09	0.36	0.08	0.44	-0.11	0.24	-0.11	0.28
Oils/Fats/Seeds	-0.15	0.12	-0.13	0.18	-0.11	0.25	0.03	0.77	0.01	0.91
Sweets/Added sugars	-0.05	0.62	-0.06	0.56	-0.02	0.85	0.00	0.98	-0.11	0.28

* Spearman correlation.

Numbers in **bold** face are statistically significant (p-value < 0.05). BMD-FN: bone mineral density of femoral neck; BMD-L1-L4: bone mineral density of lumbar spine; BMD-TF: bone mineral density of total femur; BSAP: bone-specific alkaline phosphatase; CTX: C-telopeptide of type 1 collagen.

The poor-quality and requiring improvements groups did not differ for BMD in the LS or femur (p > 0.05; Table 4 ). Both individuals with normal BD and those with osteopenia or osteoporosis presented mean dietary scores compatible with poor quality without differences between groups (48.2 ± 12.7, 49.1 ± 12.1 and 49.2 ± 10.8, respectively; p = 0.934). No significant associations were observed between dietary groups and BMD (p > 0.05; Table 5 ).

## DISCUSSION

Considering the increase in life expectancy worldwide ( 1 ), the higher risk of osteoporosis in more advanced age groups ( 3 ), and the possible influence of nutrient combinations in certain dietary patterns on bone tissue ( 29 , 30 ), the aim of this study was to verify whether there was a relationship between diet quality and bone health in a group of elderly Brazilian women, having found an association between poor diet quality and higher skeletal remodelling.

In general, the diet of all the individuals studied was found to be extremely compromised with no one presenting a good quality diet, while more than half the cases presented a poor quality diet. Diet quality is a fundamental aspect to be evaluated in an individual’s dietary practice, as it could present a risk factor for developing chronic diseases such as osteoporosis ( 8 - 10 , 31 ). Consumption of foods linked to good diet quality have in turn been linked to improved bone health, with increased dairy product and fruit intake associated with a reduced risk of osteoporosis ( 12 ). This obviously raises the question of how much the isolated consumption of dairy products, which are rich in calcium, would influence these results. However, a study with Scottish women observed that a fruit and vegetable rich diet, which are known to not be the main sources of the mineral, were associated with a reduction in bone resorption ( 11 ). In fact, healthy dietary patterns with adequate intake of fruit, vegetables, nuts and seeds seem to be positively associated with BMD ( 8 - 10 ). The mechanisms involved in this effect can be diverse. Fruit and vegetables, for example, have important nutrients for bone tissue, including minerals and vitamins. Some minerals contribute to acid-base balance, preventing bone loss, and increasing calcium retention by the kidneys, while some vitamins can help bone health by their antioxidant actions, suppressing osteoclastic activity and assisting in osteoblastic differentiation and collagen formation ( 32 ). However, it is undeniable that dairy derivatives have advantages over other foods, since, in addition to the high content of highly bioavailable calcium ( 33 ), they are also rich in magnesium, vitamins, and proteins, essential for the formation of the matrix and adequate bone mineralization ( 32 ). Unfortunately, in this study, the poorest diet was clearly associated with low consumption of these food groups, which could have negatively affected bone remodelling.

It is true that, despite existing reports linking bone health and diet quality, as well as poorness of the latest being observed in different population groups throughout the world ( 34 - 38 ), this association is still somewhat controversial, with disparate results from different authors ( 13 ). One of the possible reasons for this may be the use of different questionnaires and generic dietary assessments since there is a lack of dietary scoring indexes that adequately consider foods that influence bone tissue ( 13 , 39 ). In this sense, Healthy Eating Indexes are promising for assessing bone health in middle-aged women ( 15 ). Another factor could be variations in the types of instrument used to evaluate bone health, with many authors giving preference to different types of evaluation by image ( 39 ). In this sense, perhaps the remodelling markers indicated earlier the impact of the diet on bone tissue. In addition, the predominant ethnicity in the different studies can be very variable and influence the results. In this study, most individuals classified themselves as white, and Caucasian tend to have lower BMD than black patients ( 40 ). However, the high rate of miscegenation in Brazil makes it difficult to assess the association between ethnicity and outcome. Also, the age groups differ considerably between studies, with few authors specifically addressing individuals at higher risk of osteoporosis, namely older women ( 37 ). Our study specifically evaluated this group and, using the HEI, observed that individuals with a poor-quality diet presented higher serum levels of bone markers, indicating greater remodelling.

Remodelling is a process of bone tissue renewal and reflects the equilibrium between bone formation and resorption. During the process of osteoblastic formation, there is an increase in the production of procollagen I aminoterminal propeptide (PINP), osteocalcin, and BSAP, while during the osteoclastic resorption, small collagen fragments are released in the blood, such as CTX for example ( 41 ). Thus, these proteins can be used as useful biomarkers, reflecting bone remodelling. In this study, we observed that worse diet quality was associated with higher levels of BSAP and CTX, indicating greater bone formation and resorption. In addition, as the formation can represent a response to resorption, higher levels of BSAP could also, indirectly, indicate greater resorption. Although the remodelling is important for skeletal renewal, when constantly exacerbated it can result in bone loss and consequent osteoporosis. So, the differences between bone marker levels seen in our groups could indicate a worse osteometabolic condition in our poor-quality diet group. In fact, poor diets or dietary patterns with little variation have been negatively associated with bone health ( 10 - 12 ), and positively with risk of fractures ( 42 ). In our study, higher BSAP levels were associated with lower milk and dairy product intake while high CTX levels were associated with higher vegetable intake. Dairy products have a nutrient combination favouring intestinal mineral absorption, while vegetables have high concentrations of oxalates and phytates which can impair calcium absorption ( 33 , 43 ). Thus, both a lack of milk and dairy products and excess vegetables could compromise calcium supply from the intestine to the bloodstream, which could be maximised when both conditions are combined, as seen in our study. The consequent drop in serum calcium levels stimulates increased synthesis and release of parathyroid hormone, which in turn promotes bone resorption to normalize calcemia, negatively influencing bone mineralisation ( 44 ).

The groups with reduced bone mass and normal BD did not differ with respect to diet quality, which could be corroborated by the observation that BMD in LS, FN and WF did not differ between patients with poor diet quality and those needing improvement. These findings differ from a study performed with Korean post-menopausal women, which observed in increased association between diet quality and BMD ( 17 ). Perhaps the lack of association found in our study is due to the relative inability of the two R24Hs to evaluate the habitual diet in the long-term. Thus, R24Hs could relate to bone remodelling markers as these infer a more instantaneous bone status. On the other hand, BMD would show the long-term response to diet. In this sense, and considering that only 34% of the patients had normal BD, one could speculate that, if the same diet was to continue under current conditions, they would suffer greater densitometric compromise and their dire consequences, fractures, in the future.

This study has some limitations. The main one being the tool used to evaluate food intake, as previously discussed. It is known the risk of underreporting when using recalls. However, an advantage of our study is that the diet was evaluated at two different times, as some studies present the limitation of the evaluation in a single moment ( 39 ). Another limitation would be the cross-sectional design of the survey, which does not allow us to define a causal relationship. Thus, some of the points discussed earlier could be reduced to speculation. However, it is important to emphasize that the objective of the study was not to establish causality, and we should not depend on the verdict of causality to correct the situation that appears to be inadequate. Other limitations would include the lack of assessment of the influence of seasonality on the results, the relatively small sample size, and the characteristics of the population studied, which consisted of a very specific group of elderly women resident in a single Brazilian city, which could limit extrapolating results for other populations. However, the rigorous selection criteria used in this study also establish a positive differential for the same one. In addition, this study has highlighted that older women with poorer quality diets are undergoing greater bone remodelling.

In conclusion, in this group of elderly Brazilian women, there was a relationship between diet quality and bone health with worse diet quality associated with higher levels of bone remodelling markers. These findings strengthen the idea that strategies aimed at proper nutritional guidance for this population at risk of adverse skeletal outcomes should be drawn up aiming to minimize the risk of future consequences from an inadequate diet.
